# Inflammatory microglia correlate with impaired oligodendrocyte maturation in multiple sclerosis

**DOI:** 10.3389/fimmu.2024.1522381

**Published:** 2025-01-14

**Authors:** J. Q. Alida Chen, Dennis D. Wever, Niamh B. McNamara, Morjana Bourik, Joost Smolders, Jörg Hamann, Inge Huitinga

**Affiliations:** ^1^ Neuroimmunology Research Group, Netherlands Institute for Neuroscience, Amsterdam, Netherlands; ^2^ Departments of Neurology and Immunology, MS Center ErasMS, Erasmus MC, University Medical Center, Rotterdam, Netherlands; ^3^ Department of Experimental Immunology, Amsterdam Institute for Immunology and Infectious Diseases, Amsterdam University Medical Center, Amsterdam, Netherlands; ^4^ Swammerdam Institute for Life Sciences, Center for Neuroscience, University of Amsterdam, Amsterdam, Netherlands

**Keywords:** multiple sclerosis, remyelination, inflammation, microglia, oligodendrocytes

## Abstract

**Introduction:**

Remyelination of demyelinated axons can occur as an endogenous repair mechanism in multiple sclerosis (MS), but its efficacy varies between both MS individuals and lesions. The molecular and cellular mechanisms that drive remyelination remain poorly understood. Here, we studied the relation between microglia activation and remyelination activity in MS.

**Methods:**

We correlated regenerative (CD163^+^) and inflammatory (iNOS^+^) microglia with BCAS1^+^ oligodendrocytes, subdivided into early-stage (<3 processes) and late-stage (≥3 processes) cells in brain donors with high or low remyelinating potential in remyelinated lesions and active lesions with ramified/amoeboid (non-foamy) or foamy microglia. A cohort of MS donors categorized as efficiently remyelinating donors (ERDs; n=25) or poorly remyelinating donors (PRDs; n=17) was included, based on their proportion of remyelinated lesions at autopsy.

**Results and discussion:**

We hypothesized more CD163^+^ microglia and BCAS1^+^ oligodendrocytes in remyelinated and active non-foamy lesions from ERDs and more iNOS^+^ microglia with fewer BCAS1^+^ oligodendrocytes in active foamy lesions from PRDs. For CD163^+^ microglia, however, no differences were observed between MS lesions and MS donor groups. In line with our hypothesis, we found that INOS^+^ microglia were significantly increased in PRDs compared to ERDs within remyelinated lesions. MS lesions, more late-stage BCAS1^+^ oligodendrocytes were detected in active lesions with non-foamy or foamy microglia in comparison with remyelinated lesions. Although no differences were found for early-stage BCAS1^+^ oligodendrocytes between MS lesions, we did find significantly more early-stage BCAS1^+^ oligodendrocytes in PRDs vs ERDs in remyelinated lesions. Interestingly, a positive correlation was identified between iNOS^+^ microglia and the presence of early-stage BCAS1^+^ oligodendrocytes. These findings suggest that impaired maturation of early-stage BCAS1^+^ oligodendrocytes, encountering inflammatory microglia, may underlie remyelination deficits and unsuccessful lesion repair in MS.

## Introduction

Multiple sclerosis (MS) is a chronic inflammatory, demyelinating, and degenerative disease of the central nervous system (CNS). Remyelination is the process by which demyelinated axons are remyelinated by oligodendrocytes. This process is crucial in MS, as myelin is required for effective conduction of axon potential in axons and remyelination would therefore restore impaired neurological function (1–3). Notably, the extent of remyelination is highly variable between individuals with MS (4) and between MS lesion subtypes (5). MS patients with a relapsing disease course have a higher proportion of remyelinated lesions compared to patients with a more progressive disease course (6). Additionally, remyelination is more commonly observed in younger patients and appears to fail with advancing age (7). Regarding MS lesion subtypes, remyelination has been observed to occur most efficiently in early, active lesions (2, 8).

There is increasing evidence for a beneficial role of microglia/macrophages (hereafter referred to as microglia) in remyelination through the production of growth factors and cytokines. These may promote recruitment of oligodendrocyte progenitor cells (OPCs) and stimulate their differentiation into mature oligodendrocytes. Microglia also aid this process through the clearance of myelin debris (9, 10), which may be controlled by their activation state (11, 12). It is thought that in the early phase of repair, pro-inflammatory microglia expressing iNOS, TNF, and CD16/CD32+ microglia help to clear myelin debris and recruit OPCs to the lesion site, while in the later phase, regenerative or anti-inflammatory microglia expressing ARG1, IGF1, CD206, and CD163 aid OPC differentiation into mature oligodendrocytes (13, 14). Microglia can also be distinguished morphologically. Ramified and amoeboid microglia (hereafter referred to as non-foamy) have been associated with homeostasis, neuroprotection, less axonal damage, and phagocytosis (15, 16), while myelin laden foamy microglia in active and mixed active/inactive lesions have been associated with a more severe disease, presence of B and T cells, and (acute) neuro-axonal damage (16, 17).

A molecular marker to visualize actively (re)myelinating oligodendrocytes is in great need, as this could give rise to specific areas, pathways, and molecules of interest to study for novel therapeutic targets. Recent studies have suggested that markers of pre-myelinating oligodendrocytes, including breast carcinoma amplified sequence 1 (BCAS1) (18–21), may serve as promising markers for early active remyelination. This marker has been shown to be expressed by oligodendrocytes in an intermediate state of differentiation from OPCs to mature oligodendrocytes (18). To our knowledge, this is the first study that makes a distinction between early- and late-stage BCAS1^+^ oligodendrocytes by assessing the amount of radiating processes in post-mortem tissue of MS donors. The distinction has been made before in relation to cerebral ischemic stroke and small vessel disease patients (20), and multiple system atrophy patients (21).

In this study, we have investigated the relationship between the activation state of microglia (pro-inflammatory iNOS^+^IBA1^+^ and regenerative CD163^+^IBA1^+^) and presence of pre-myelinating (BCAS1^+^) oligodendrocytes using immunohistochemical analyses. Additionally, by subclassifying BCAS1^+^ oligodendrocytes into early- and late-stage cells, we explored whether specific BCAS1^+^ cell maturation stages in the remyelination process differentiate efficiently from poorly remyelinating MS donors and lesions. Our findings show that the density of early-stage BCAS1^+^ oligodendrocytes is associated with abundance of pro-inflammatory iNOS^+^ microglia and that the density of these early-stage BCAS1^+^ cells is increased in brain donors with a poor remyelinating capacity compared to efficiently remyelinating donors. Taken together, this suggests that not a lack of OPCs but rather the incomplete maturation of OPCs leads to remyelination failure in the presence of pro-inflammatory microglia in MS. It is crucial to investigate the underlying mechanisms that restrict successful oligodendrocyte maturation to promote efficient remyelination in future therapies.

## Materials & methods

### Post-mortem brain tissue

Human brain samples were provided by the Netherlands Brain Bank (NBB). Informed consent was obtained from all donors for the use of material and clinical data for research purposes. The NBB autopsy procedures were approved by the Ethics Committee of Amsterdam UMC, location VUmc, Amsterdam, The Netherlands. Post-mortem formalin-fixed and paraffin-embedded (FFPE) brain samples of 42 MS donors were collected from subcortical MS lesions. A total of 34 remyelinated lesions, 22 active non-foamy lesions, and 24 active foamy lesions were included. Sections with suboptimal staining quality or those which were no longer available were excluded from the analysis of the respective marker. For BCAS1 analysis, 2 remyelinated lesion, 1 active non-foamy lesion, and 2 active foamy lesion samples from a total of 4 donors were excluded. For iNOS/IBA1, 1 active foamy lesion sample was excluded from 1 donor. Lastly, for CD163/IBA1, 5 remyelinated lesions, 2 active non-foamy lesions, and 1 active foamy lesion from 8 donors were excluded.

### Lesion classification and tissue dissection

MS lesion types were classified based on the degree of demyelination and innate inflammatory lesion activity as previously described (6, 22). Double immunostaining was performed to visualize human leukocyte antigen (HLA-DR-DP-DQ, referred to as HLA) (Ab7856, Abcam, Cambridge, UK), with diaminobenzidine (DAB)-nickel) and proteolipid protein (PLP) (MCA839G; Serotec, Oxford, UK, with DAB). Remyelinated lesions displayed pale staining intensity of PLP and sparse HLA^+^ microglia, and active lesions displayed loss of PLP myelin protein and accumulation of HLA^+^ cells throughout the lesion. Active lesion subtypes were classified based on the morphology of microglia within the lesions. Remyelinated and active non-foamy lesion types were considered to be associated with remyelination, and active foamy lesions were considered to be negatively associated with myelin repair, based on a positive correlation with the proportion of remyelinated lesions per donor as shown previously (23).

### Donor selection

Neurological diagnosis of MS was confirmed post-mortem by a certified neuropathologist. The proportion of remyelinated lesions was calculated by dividing the amount of remyelinated lesions by all remyelinated + inactive lesions. Lesion load (log(x + 1)) from standardly dissected brainstem tissue was calculated and transformed as previously established (6). MS donors were stratified into two donor subgroups based on the proportion of remyelinated lesions as done previously (23). We previously assessed the proportion of remyelinated lesions in every NBB MS donor from 1990-2020. From a total of 239 donors, the median of proportion of remyelinated lesions was 0.27 (23). MS donors with a proportion of remyelinated lesions >0.27 were considered efficiently remyelinating donors (ERDs), and those <0.27 were considered poorly remyelinating donors (PRDs). A total of 25 ERDs and 17 PRDs were included in this study. Characteristics of all included MS brain donors are displayed in **Supplementary Table 1**.

### BCAS1 morphological classification

All BCAS1 immuno-positive cells were quantified and classified as “total BCAS1^+^ cells”. As BCAS1^+^ oligodendrocytes have been observed to acquire a more complex morphology with multiple branched processes during the process of differentiation (18, 20), the number of processes were used as a measure to distinguish early- versus late-stage BCAS1^+^ oligodendrocytes, similar to a previous studies on donors with multiple system atrophy (21) and donors with cerebral ischemic stroke and small vessel disease (20). Here, BCAS1^+^ cells with <3 processes were considered early-stage and those with >3 processes were considered late-stage oligodendrocytes.

### Immunohistochemistry

FFPE human brain sections of 8 μm were deparaffinized and rehydrated in a series of xylene and ethanol concentrations. Antigen retrieval was performed by microwaving at 700 W for 10 min in citrate buffer pH6.0 or Tris-EDTA buffer pH9.0. All sections were blocked with blocking buffer [10% normal horse serum + 1% bovine serum albumin (BSA) + 0.5% Triton X-100 in Tris-buffered saline (TBS) pH7.6]. Endogenous peroxidase activity was blocked with 1% H_2_O_2_ and 0.2% Triton-X-100 in TBS. Primary antibodies were incubated overnight at 4°C. For immunohistochemistry, BCAS1 (Sc-136342, 1:30,000, Santa Cruz) was included. Sections were incubated with biotinylated anti-mouse secondary antibody for 1 h, followed by incubation with avidin-biotin complex (ABC) kit for 45 min (PK-6100, 1:800, Vector Laboratories). Sections were visualized with the DAKO REAL Envision detection kit (K500711-2, 1:100, DAKO), and subsequently counterstained with hematoxylin and dehydrated.

For fluorescent double-labeling, IBA1 (019-19741, 1:750, WAKO) was used as a microglia/macrophage marker. Polarization to regenerative or pro-inflammatory states was defined using CD163 (NB110-40686, 1:200, Novus Biologicals) and iNOS/NOS type II (610328, 1:200, BD Biosciences), respectively. The specific staining conditions of all primary antibodies are summarized in **Table 1**. For detection of IBA1, a fluorescent-labeled anti-rabbit secondary antibody conjugated with Alexa Fluor™ 488 was incubated for 1 h at room temperature (RT). For CD163 and iNOS, sections were incubated with appropriate biotinylated secondary antibodies, followed by incubation with ABC-kit, as above. Additional signal enhancement for iNOS was achieved by incubation with biotinylated tyramide (1:10,000 in PBS with 0.001% H_2_O_2_) for 10 min. After a second ABC incubation, the sections were incubated with a streptavidin-conjugated with Cy3 fluorophore for 1 h at RT. All sections were counterstained with Hoechst 33342 (H3570, 1:1,000; Thermo Fisher Scientific) for 10 min and incubated with 0.1% Sudan Black in 70% ethanol for 5 min, before embedding with mounting medium [0.605 g Tris, pH8.5 + 12.5 ml glycerol + 5 g Mowiol (EMD Chemicals, Gibbstown, NJ, USA)].

**Table 1:** Antibodies used for immunohistochemistry.

Antibody	Company	Category number	Host	Clonality	Dilution	Antigen retrieval
BCAS1/NaBC1	Santa Cruz	Sc-136342	Mouse	Monoclonal	1:30,000	Citrate buffer pH 6.0
PLP	BioRad	MCA839G	Mouse	Monoclonal	1:1500	TBS pH 7.6
HLA	Abcam	Ab7856	Mouse	Monoclonal	1:100	TBS pH 7.6
Iba1	WAKO	019-19741	Rabbit	Polyclonal	1:750	Tris-EDTA pH 9.0
iNOS/NOS type II	BD Biosciences	610328	Mouse	Monoclonal	1:200	Tris-EDTA pH 9.0
CD163 (EDHu-1)	Novus Biologicals	NB110-40686	Mouse	Monoclonal	1:200	Tris-EDTA pH 9.0

### Quantification of immunohistochemistry

All images were acquired at 20x magnification using a Zeiss Axio Scan.Z1 slide scanner (Carl Zeiss, Oberkochen, Germany). Regions of interest from lesioned areas of BCAS1, iNOS/IBA1, and CD163/IBA1 stainings were determined using adjacent HLA-PLP sections. Positive cells were detected using a random trees-based classifier. From all BCAS1^+^ cells identified through this positive-cell detection method, late-stage BCAS1^+^ oligodendrocytes were manually subclassified. Data were analyzed using QuPath (v0.4.0) and processed using the Fiji plugin for ImageJ software.

### Statistical analyses

Differences in demographical and clinical characteristics were tested using the Kruskal-Wallis rank sum test for comparing continuous variables across groups and the Chi-square test for comparing categorical variables. Differences in proportions of remyelinated lesions were determined with the quasi-binomial generalized linear model (GLM). Numbers of BCAS1^+^ cells, CD163^+^IBA1^+^ cells, and iNOS^+^IBA1^+^ cells per mm^2^ were tested using the negative binomial GLM with offset by the area measured, and corrected for donors. Data are represented as plots showing the mean data points of similar lesions from the same donor (average data point per lesion). Plots showing estimated marginal means were used for statistical analyses. Samples Tukey’s *post-hoc* test was performed to compare multiple MS donor and tissue groups. Correlations were tested using Pearson’s correlation coefficient with Benjamini-Hochberg correction for multiple testing. All analyses were performed in R (v4.1.0) using the lme4, glmmTMB, emmeans and stats packages. *P*-values <0.05 were considered to be statistically significant.

## Results

### Donor demographical and clinical characteristics

This study included a total of 42 donors (ERDs, n=25; PRDs, n=17). Donor selection based on remyelination potential was performed by assessing the proportion of remyelinated lesions at autopsy. As expected, the sum of remyelinated lesions (± SD) as well as the average proportion of remyelinated lesions (± SD) was higher for ERDs (9.9 ± 11.5 and 0.62 ± 0.23) compared to PRDs (2.4 ± 2.3 and 0.11 ± 0.08) (*p*<0.001 and *p*<0.0001, respectively) (**Table 2**). The donor groups were matched for age, post-mortem delay, sex, pH of the cerebrospinal fluid, and brain weight. No differences were detected for age at disease onset, disease duration, and years to expanded disability status scale 6. However, PRDs had a significantly higher lesion load compared to ERDs (*p*=0.050), reflective of a worse clinical course.

**Table 2:** Demographic and clinical characteristics of selected cohort.

	All donors (N=42)	All ERDs (n=25)	All PRDs (n=17)	P-value
**Disease subtype**	4 R-MS 17 PPMS 18 SPMS 3 unknown	4 R-MS 12 PPMS 8 SPMS 1 unknown	0 R-MS 5 PPMS 10 SPMS 2 unknown	
**Number of lesions**	34 RL 22 AL non-foamy 24 AL foamy	21 RL 13 AL non-foamy 11 AL foamy	13 RL 9 AL non-foamy 13 AL foamy	
**Proportion of remyelinated lesions**	0.41 (0.31)	0.62 (0.23)	0.11 (0.08)	***p*<0.0001** ^i^
**Sum of remyelinated lesions**	6.8 (9.7)	9.9 (11.5)	2.4 (2.3)	***p<*0.001** ^iii^
**Age (years)**	59.9 (11.3)	62.5 (10.9)	56.1 (11.1)	*p*=0.066^iii^
**PMD (minutes)**	515 (121)	521 (111)	505 (138)	*p*=0.798^iii^
**Sex (F%)**	64%	64%	65%	*p*=1^ii^
**pH of CSF**	6.5 (0.3)	6.5 (0.2)	6.4 (0.4)	*p*=0.308^iii^
**Brain weight (grams)**	1180 (124)	1177 (132)	1184 (113)	*p*=0.749^iii^
**Age at onset (years)**	35.9 (10.6)	36.2 (10.1)	35.4 (11.5)	*p*=0.701^iii^
**Disease duration (years)**	24.2 (11.7)	26.8 (12.5)	20.7 (9.8)	*p*=0.162^iii^
**Years to EDSS6 (years)**	14.0 (10.6)	15.9 (12.3)	11.7 (7.6)	*p*=0.462^iii^
**Lesion load (standardized)**	17.5 (14.1)	15.7 (16.3)	20.2 (9.8)	***p*=0.050** ^iii^

Data presented as mean (SD), unless otherwise indicated. ERD, efficiently remyelinating donor; PRD, poorly remyelinating donor; PMD, post-mortem delay; CSF, cerebrospinal fluid; RL, remyelinated lesion; AL non-foamy, active ramified and amoeboid lesion; AL foamy, active foamy lesion; R-MS, relapsing MS; PPMS, primary progressive MS; SPMS, secondary progressive MS; EDSS, expanded disability status scale. Bold values indicate significant *p-*values. ^i^Quasi-binomial generalized linear model; ^ii^Chi-square test; ^iii^Kruskal-Wallis test.

### Pro-inflammatory microglia are enriched in poorly remyelinating donors compared to efficiently remyelinating donors

**Figure 1:**
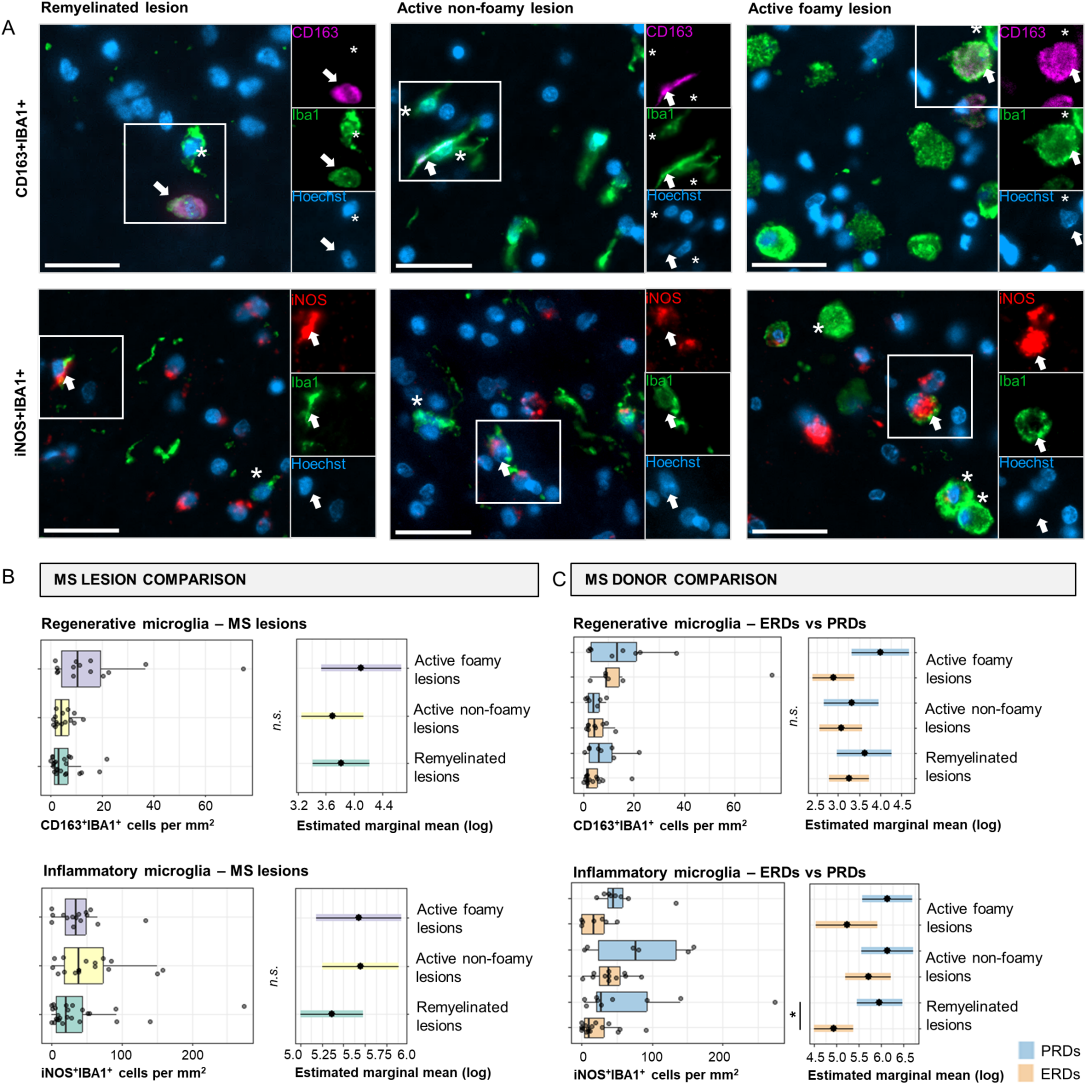
Microglia activation state comparisons in MS lesion types and donor groups. **(A)** Immunofluorescent double-stained images of CD163^+^ (regenerative) and iNOS^+^ (pro-inflammatory) with IBA1^+^ (microglia) in a remyelinated lesion, active non-foamy lesion, and active foamy lesion. Scale bar indicates 30 µm. Arrows indicate double-positive cells. Asterisks indicate CD163^-^IBA1^+^ or iNOS^-^IBA1^+^ cells. **(B)** Histological quantification of CD163^+^IBA1^+^ and iNOS^+^IBA1^+^ cells in remyelinated lesions, active non-foamy lesions, and active foamy lesions. **(C)** Histological quantification of CD163^+^IBA1^+^ and iNOS^+^IBA1^+^ cells per mm^2^ compared between ERDs and PRDs. Statistics were performed using negative binomial GLM test with Tukey’s *post-hoc* correction to compare between groups. Estimated marginal means reflect predicted mean of statistical model, with adjustment for multiple datapoints (lesions) per donor. *p<0.05; **p<0.01; ***p<0.001. N.s., not significant.

First, the presence of microglia with a regenerative (CD163^+^IBA1^+^) or pro-inflammatory (iNOS^+^IBA1^+^) activation state (13, 14) was assessed in different MS lesion (sub)types (**Figure 1**). No differences were found for the number of CD163^+^IBA1^+^ or iNOS^+^IBA1^+^ cells between MS lesion (sub)types (**Figure 1B**). Next, we assessed whether expression of iNOS and CD163 of IBA1^+^ cells in remyelinated, active non-foamy, and active foamy lesions were different in ERDs and PRDs (**Figure 1C**). Interestingly, while no significant difference was observed between ERDs and PRDs in number of CD163^+^IBA1^+^ cells, we found a significantly higher number of iNOS^+^IBA1^+^ cells in PRDs in comparison to ERDs in remyelinated lesions (*p*=0.035). Together, this implies that a more pro-inflammatory microglia phenotype, rather than a regenerative phenotype, distinguishes between remyelination failure and success in different MS donors.

### Oligodendrocyte stage-dependent expression of BCAS1 in MS lesion types

**Figure 2:**
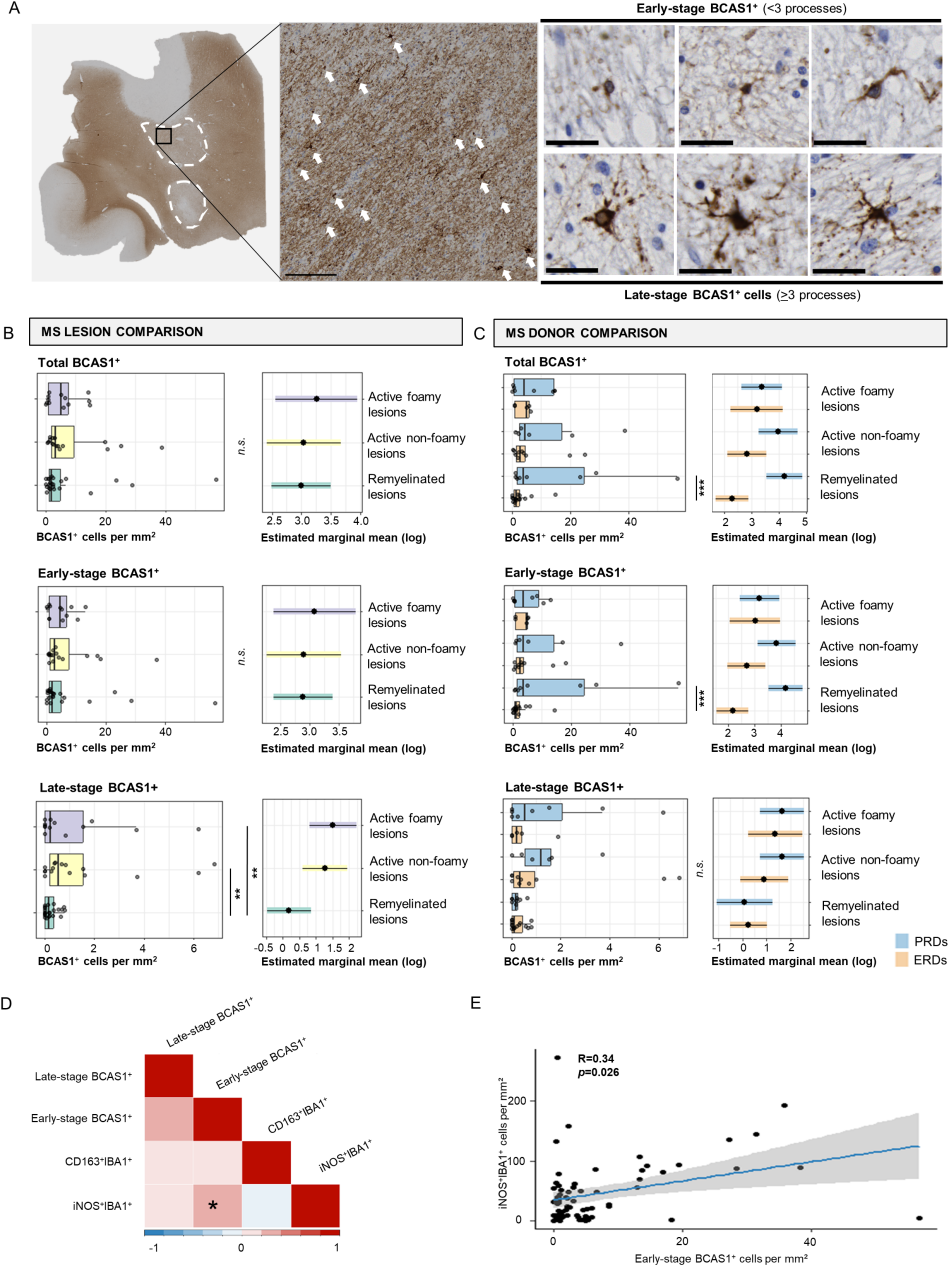
Early- and late-stage BCAS1^+^ cell comparisons in MS lesions and donors and association with microglia activation states. **(A)** Immunohistochemical image of BCAS1 staining in an active non-foamy lesion, and classification of early-stage versus late-stage BCAS1^+^ oligodendrocytes. White arrows indicate BCAS1^+^ cells. Scale bar in overview image indicates 150 µm. Scale bar of early- and late-stage BCAS^+^ oligodendrocyte classification indicates 30 µm. **(B)** Histological quantification of total BCAS1^+^, early-stage BCAS1^+^, and late-stage BCAS1^+^ cells per mm^2^ in remyelinated, active non-foamy, and active foamy lesions, and **(C)** compared between ERDs and PRDs. **(D)** Correlations between the number of early-stage BCAS1^+^ oligodendrocytes, late-stage BCAS1^+^ oligodendrocytes, CD163^+^IBA1^+^ microglia, and iNOS^+^IBA1^+^ microglia per mm^2^ shows **(E)** a positive correlation between iNOS^+^IBA1^+^ microglia per mm^2^ and early-stage BCAS1^+^ oligodendrocytes. Statistics were performed using negative binomial GLM test with Tukey’s *post-hoc* correction to compare between groups. Estimated marginal means reflect predicted mean of statistical model, with adjustment for multiple datapoints (lesions) per donor. Correlations were tested using Pearson’s correlation coefficient with Benjamini-Hochberg for multiple testing correction. *p<0.05; **p<0.01; ***p<0.001. N.s., not significant.

To validate expression of BCAS1 by pre-myelinating oligodendrocytes, we performed datamining of single-cell/nucleus transcriptomic datasets of mouse CNS (24) and human MS normal-appearing white matter, active, chronic active, chronic inactive and remyelinated lesions, and control white matter samples (25). Marques et al. (24) showed that *Bcas1* is mainly expressed by committed oligodendrocyte precursors (COP) and newly formed oligodendrocytes (NFOL) in mice CNS (**Supplementary Figure 1A**). Jäkel et al. (25) showed that in oligodendrocyte lineage cells in human white matter, similar to mice, *BCAS1* is mainly expressed by oligodendrocyte precursor cells (OPCs) and COPs, as well as by the Oligo1 subcluster (**Supplementary Figure 1B**). Although Oligo1 was largely made up of mature oligodendrocytes, this cluster was depleted in MS compared to control tissue (25). These studies confirm that *BCAS1* is mainly expressed in cells at an intermediate stage between OPCs and mature oligodendrocytes, early in oligodendrocyte differentiation (18). Thus, BCAS1 expression was used as a marker for early active remyelination in remyelinated, active non-foamy, and active foamy lesions in our cohort. We analyzed total, early- and late-stage BCAS1^+^ oligodendrocytes through classification of the number of branched processes of BCAS1^+^ oligodendrocytes (**Figure 2A**). In general, BCAS1^+^ cells were predominantly early-stage oligodendrocytes with few branched processes, rather than late-stage oligodendrocytes with >3 processes, as shown by the quantitative analyses of total, early- and late-stage BCAS^+^ oligodendrocytes (**Figure 2B**). Numbers of both total and early-stage BCAS1^+^ oligodendrocytes did not differ between MS lesion subtypes. However, the number of late-stage BCAS1^+^ oligodendrocytes was higher in active lesions with both non-foamy and foamy microglia compared to remyelinated lesions (*p*=0.010 and *p*=0.009, respectively) (**Figure 2B**).

### Increased numbers of BCAS1^+^ early-stage oligodendrocytes in donors with poor remyelination compared to donors with efficient remyelination

Subsequently, we compared the presence of early- and late-stage BCAS1^+^ oligodendrocytes between the donor groups with different remyelination capacity (**Figure 2C**). When comparing ERDs and PRDs, we observed an increased number of total and early-stage BCAS1^+^ cells in remyelinated lesions of PRDs in comparison with ERDs (*p*<0.001 and *p*<0.001, respectively). The total number of late-stage BCAS1^+^ cells showed no difference between ERDs and PRDs (**Figure 2C**). Since the increase in BCAS1^+^ oligodendrocytes in PRDs compared to ERDs is mainly accounted for by BCAS1^+^ oligodendrocytes in their early-stage, this indicates that the cells that potentially promote remyelination are present in donors with poor remyelination capacity early in the repair process. Yet remyelination ultimately fails.

### iNOS^+^ microglia correlate with more early-stage BCAS1^+^ oligodendrocytes

To determine the relationship between microglia activation state and BCAS1^+^ oligodendrocytes, we correlated the number of BCAS1^+^ (late-stage BCAS1^+^ and early-stage BCAS1^+^) oligodendrocytes with the number of CD163^+^IBA1^+^ and iNOS^+^IBA1^+^ cells (**Figure 2D**). Remarkably, only iNOS^+^IBA1^+^ showed a positive correlation with early-stage BCAS1^+^ cells (*p*=0.026) (**Figures 2D, E**). No correlation was further found between early- and late-stage BCAS1^+^ oligodendrocytes. Additionally, no correlation was identified for CD163^+^IBA1^+^ cells with different maturation stages of BCAS1^+^ oligodendrocytes. Together, these findings show that pro-inflammatory microglia are associated with early-stage BCAS1^+^ oligodendrocytes.

## Discussion

Here, we explored the relationship between microglia activation and remyelination activity in MS donors and lesions with different remyelinating capability using post-mortem human brain tissue. We found an increase in number of pro-inflammatory iNOS^+^ microglia and early-stage BCAS1^+^ oligodendrocytes in PRDs vs. ERDs remyelinated lesions. Furthermore, a positive correlation was identified between iNOS^+^ microglia and the presence of early-stage BCAS1^+^ oligodendrocytes. No significant differences between MS lesion types or between MS donor groups were further detected for the number of regenerative CD163^+^ microglia. These findings suggest that impaired maturation of early-stage BCAS1^+^ oligodendrocytes, encountering inflammatory iNOS+ microglia, may underlie remyelination deficits and unsuccessful lesion repair in MS.

Previously, it was shown that microglia regulate remyelination at multiple stages, including OPC recruitment and proliferation, oligodendrocyte differentiation, and clearance of myelin debris (9, 14, 26, 27), which may be directed by the microglia activation state (11, 12). We found a positive relationship between increased pro-inflammatory microglia and early-stage BCAS1^+^ oligodendrocytes, but not late-stage BCAS1^+^ oligodendrocytes. This finding suggests that iNOS+ microglia may inhibit OPC maturation. Indeed, pro-inflammatory microglia promote remyelination in the early phase through OPC proliferation and migration *in vivo* (14).

Studies have shown that BCAS1 is co-expressed with the pan-oligodendroglial markers OLIG2 and SOX10 (18), but rarely overlaps with the OPC marker NG2, or the mature oligodendrocyte markers CC1 or TPPP/p25 (18, 20) using immunohistochemistry. This further suggests that BCAS1 is expressed by oligodendrocytes at an intermediate stage of development. *In vitro* studies have also showed that during the early stage of differentiation, BCAS1^+^ cells appear rounder with almost no branched processes, while in the later stage, the cells acquire an arborized morphology (18, 20). From all BCAS1^+^ oligodendrocytes, only small numbers were late-stage cells, as detected in our study and others (20). Our findings pointed toward an increased number of late-stage BCAS1^+^ cells in both active lesion subtypes compared to remyelinated lesions, while no differences were observed in number of early-stage BCAS1^+^ cells. These findings are in line with the theory that active remyelination can occur in active lesions, while the remyelination process has already been completed in remyelinated lesions (i.e. shadow plagues) (28). Additionally, these findings are in line with single-nucleus transcriptomic data showing that OPCs and COPs, the cells that primarily express BCAS1, are reduced in remyelinated lesions compared to active lesions (25).

When comparing ERDs with PRDs, our results showed a higher number of early-stage BCAS1^+^ oligodendrocytes in PRDs compared to ERDs in remyelinated lesions. However, no significant differences were observed between the donor groups regarding the number of late-stage BCAS1^+^ cells. Together, this suggests that the potential of remyelination is present in early stages of repair in PRDs, but that this may not result in actual remyelination.

Previously, foamy microglia have been associated with more severe disease and greater axonal damage (16, 17), while non-foamy microglia have been associated with homeostasis, neuroprotection, reduced axonal damage, and phagocytosis (15, 16). However, our findings showed that microglia morphology and myelin uptake did not impact on expression of CD163^+^ and pro-inflammatory iNOS^+^, or BCAS1^+^ oligodendrocyte maturation stage.

Previously, it was found that patients with stroke showed an increase in number of early-stage BCAS1^+^ oligodendrocytes, but not in late-stage BCAS1^+^ oligodendrocytes, compared to control donors without CNS pathology (20). In this study, authors concluded that remyelination dysfunction could be attributed to insufficient maturation of BCAS1^+^ oligodendrocytes in stroke. As only one donor in our cohort had a confirmed history of stroke, it is unlikely that this affected the results of our study.

There is increasing evidence for heterogeneity in oligodendrocyte populations, showing distinct transcriptional profiles, including MS mouse models with disease-associated (SERPINA3) signature of oligodendrocytes (25, 29–32). It would be of interest to determine whether there are differences in the transcriptional state of early-stage BCAS1^+^ oligodendrocytes, for instance in relation to immunity, senescence, stress or apoptosis markers, to understand the molecular mechanisms underpinning remyelination failure in ALs foamy of PRDs.

A limitation of this study is the use of post-mortem human brain tissue, showing results at one cross-sectional time point. As lesion evolution and remyelination are dynamic processes (1, 33), it could be that temporal changes are missed. Nonetheless, our study provides significant insight and a strong basis to begin understanding why remyelination fails in MS donors and lesions with poor remyelination capability. Further research, such as functional analyses on remyelination capacity in the context of iNOS-expressing microglia-oligodendrocyte interactions, should be performed to build on and extend our findings.

In summary, we found that BCAS1 is associated with pro-inflammatory microglia, and that these early-stage BCAS1^+^ oligodendrocytes are increased in donors with poor remyelination capability. These findings implicate incomplete oligodendrocyte maturation as a key issue underpinning failure of remyelination in MS. We believe that BCAS1 may predominantly be used as a marker of remyelination in an early phase, and that discrimination between early- and late-stage BCAS1^+^ oligodendrocytes, may help to capture later phases of remyelination processes.

## Data Availability

The raw data supporting the conclusions of this article will be made available by the authors, without undue reservation.

## Supplementary material

Supplementary Figure 1 Datamining of transcriptomic datasets for cellular expression of *BCAS1*. Single-cell/nucleus transcriptomic dataset of **(A)** mouse CNS (adapted from Marques et al., 2018) and **(B)** human MS and control brain samples (adapted from Jäkel et al., 2019). COP(s), committed oligodendrocyte precursor(s); NFOL, newly formed oligodendrocytes; OPCs, oligodendrocyte precursor cells.
