# Supplementary material for: Inflammatory microglia correlate with impaired oligodendrocyte maturation in multiple sclerosis
Source: Front Immunol. 2025 Jan 14;15:1522381. doi: 10.3389/fimmu.2024.1522381 (PMC11772157; doi:10.3389/fimmu.2024.1522381)
Supplement: Supplementary file 2 [file Table1.docx]

**Supplementary Table 1. Donor demographics and characteristics**

| **Donor#** | **Donor group** | **RL proportion** | **Sex** | **Age** | **pH CSF** | **Pmd (min)** | **Disease duration (years)** | **MS type** | **Quantified lesions (n)** | **Cause of death** |
| --- | --- | --- | --- | --- | --- | --- | --- | --- | --- | --- |
| D1 | ERD | 1 | F | 69 | 6.4 | 555 | 25 | SP | 1 AL non-foamy | General decline due to MS |
| D2 | ERD | 0.5 | F | 62 | 6.49 | 405 | 25 | PP | 1 AL non-foamy, 1 AL foamy | Cardiac asthma |
| D3 | ERD | 0.67 | F | 82 | 6.38 | 270 | 22 | PP | 1 RL | Cachexia |
| D4 | ERD | 1 | F | 79 | 6.52 | 600 | 44 | PP | 1 RL | Cardiac arrest |
| D5 | ERD | 0.31 | M | 64 | 6.84 | 450 | 34 | PP | 1 RL, 1 AL non-foamy | End stage progressive MS |
| D6 | ERD | 0.5 | M | 81 | 6.33 | 530 | 51 | PP | 1 RL, 1 AL foamy | General deterioration |
| D7 | ERD | 0.49 | F | 48 | 6.64 | 350 | 22 | RR | 4 RLs | Congestive cardiac failure |
| D8 | ERD | 0.47 | F | 68 | 6.3 | 450 | 39 | PP | 2 RLs | Bronchitis/ aspiration pneumonia |
| D9 | ERD | 0.51 | F | 68 | 6.9 | 470 | 17 | PR | 2 RLs | Pneumonia |
| D10 | ERD | 0.6 | F | 50 | 6.66 | 465 | 17 | PP | 1 AL non-foamy | Euthanasia |
| D11 | ERD | 0.31 | F | 44 | 6.34 | 615 | 16 | PP | 1 RL | Decompensation |
| D12 | ERD | 0.5 | M | 47 | 6.2 | 435 | 7 | SP | 1 AL foamy | Urosepsis with organ failure |
| D13 | ERD | 0.77 | F | 48 | 6.21 | 700 | 23 | SP | 1 RL | Hepatic encephalitis |
| D14 | ERD | 0.75 | M | 71 | 6.6 | 420 | 27 | PP | 1 AL non-foamy | Pneumonia |
| D15 | ERD | 0.59 | F | 68 | 6.4 | 640 | 42 | SP | 1 RL, 2s AL non-foamy | Euthanasia |
| D16 | ERD | 0.38 | M | 51 | 6.23 | 660 | NA | SP | 3 ALs foamy | NA |
| D17 | ERD | 0.5 | F | 60 | 6.48 | 640 | 7 | SP | 1 AL foamy | Euthanasia |
| D18 | ERD | 0.67 | M | 54 | 6.39 | 495 | 14 | PP | 1 AL non-foamy | Euthanasia |
| D19 | ERD | 1 | F | 66 | 6.45 | 575 | 32 | PP | 2 ALs non-foamy | Euthanasia |
| D20 | ERD | 0.36 | M | 67 | NA | 660 | 38 | PR | 1 RL, 1 AL non-foamy | Sudden death |
| D21 | ERD | 1 | F | 66 | 6.73 | 645 | 19 | RR | 1 RL, 1 AL non-foamy, 1 AL foamy | Pulmonary hypertension |
| D22 | ERD | 0.36 | M | 54 | 6.54 | 400 | 30 | PP | 1 RL, 1 AL non-foamy | Euthanasia |
| D23 | ERD | 0.67 | F | 61 | 6.88 | 600 | NA | NA | 1 RL, 3 ALs foamy | Euthanasia |
| D24 | ERD | 0.59 | F | 74 | 6.4 | 470 | 49 | SP | 1 RL | Euthanasia |
| D25 | ERD | 1 | M | 60 | 6.67 | 529 | 17 | SP | 1 RL | Euthanasia |
| D26 | PRD | 0 | F | 40 | 6.33 | 420 | 11 | SP | 2 ALs foamy | Pneumonia |
| D27 | PRD | 0 | F | 45 | 6.62 | 655 | 14 | SP | 1 AL foamy | Euthanasia |
| D28 | PRD | 0.19 | M | 51 | 6.36 | 470 | 29 | SP | 2 RLs | Uremia and cachexia |
| D29 | PRD | 0 | F | 48 | 6.55 | 490 | 9 | SP | 1 AL non-foamy, 3 ALs foamy | Euthanasia |
| D30 | PRD | 0 | M | 77 | 6.25 | 255 | 32 | PP | 1 AL foamy | Cerebral vascular accident |
| D31 | PRD | 0.12 | F | 48 | 6.63 | 290 | 25 | PP | 1 RL, 1 AL non-foamy | Euthanasia |
| D32 | PRD | 0.17 | M | 53 | 6.7 | 330 | 2 | PP | 1 AL foamy | Respiratory insufficiency |
| D33 | PRD | 0.18 | F | 57 | 6.05 | 520 | 27 | SP | 1 RL, 2 ALs non-foamy | Respiratory insufficiency |
| D34 | PRD | 0.15 | F | 57 | 6.44 | 520 | 29 | NA | 3 RL, 2 ALs non-foamy | Euthanasia |
| D35 | PRD | 0.11 | M | 54 | 6.26 | 650 | 27 | SP | 2 ALs non-foamy | Euthanasia |
| D36 | PRD | 0.15 | F | 48 | 6.1 | 710 | 22 | SP | 1 RL | Respiratory failure |
| D37 | PRD | 0.1 | F | 54 | 6.61 | 565 | 27 | SP | 2 ALs foamy | Respiratory failure |
| D38 | PRD | 0.11 | M | 48 | 6.29 | 395 | 16 | SP | 2 ALs foamy | Ileus and dehydration |
| D39 | PRD | 0.21 | F | 61 | 5.9 | 655 | 11 | PP | 1 AL foamy | Euthanasia |
| D40 | PRD | 0.21 | F | 61 | 5.9 | 655 | 11 | PP | 1 RL | Sepsis |
| D41 | PRD | 0.13 | F | 81 | 7.35 | 440 | 21 | NA | 2 RLs | Dehydration |
| D42 | PRD | 0.06 | M | 70 | 7.22 | 565 | 38 | SP | 2 RLs, 1 AL non-foamy | Euthanasia |

CSF = cerebrospinal fluid; ERD = efficiently remyelinating donor; PRD = poorly remyelinating donor; F = female; M = male; PMD = post-mortem delay; R-MS = relapsing MS; PPMS = primary progressive MS; NA = not available; RL = remyelinated lesion; AL non-foamy = active ramified and amoeboid lesion; AL foamy = active foamy lesion. RL proportion indicates the proportion of RLs from all RLs and inactive lesions at autopsy.
